# In the Children's Ward

**Published:** 1887-03-12

**Authors:** Walter J. Eccott


### IN THE CHILDREN'S WARD.

By Walter J. Eccott.

### Chapter II.

The children's ward at "St. Elizabeth's" was beautiful. At first sight it struck a new comer as not being at all like a hospital ward. And why should hospital wards present a dreary aspect? What was it made this ward look so different from many? Because the walls were bright. The brightness was not that produced by a dead white or grey surface, simply relieved by a thin coloured line, and breaking out, as it were, in a rash of gaudily coloured texts, which present more or less of a puzzle to little children, and perhaps often more weariness than consolation to older patients. The walls themselves were beautiful. A high dado of dark, glossy chocolate colour stretched around the ward, and on this background, with those touches of life and nature which only a true artist can impart, were painted flowers of the greatest and most varied loveliness. Not here were the cold, conventional flowers of needlework and cup-and-saucer art, but cornflowers, blue and white, springing up between golden wheat-ears, narcissi, lilies, roses and great yellow daffodils. All looked natural and fresh and bright, seeming to spring out of the very floor as from a fertile garden-soil. No contract work this, but a labour of love. As you gazed upon it you could not help thinking of Fra Angelico, and how he adorned with masterpieces the walls of dormitories and refectory in the monastery of San Marco, at Florence; not because he was bidden, nor with any thought of personal renown, but that he could conceive no greater glory than to consecrate the powers that God had lent him to beautifying the house of His servants. Is a hospital less deserving than a monastery of such self-abnegation? What if a few more artists were to devote some of their time to this unpaid, inglorious work? It would not be labour lost. An artist paints a picture; it is bought and hung, probably, in the house of some wealthy man, whose own and few friends' eyes criticise or feast upon it. After a certain time who looks at it? How much better that the same expenditure of toil, the same wealth of love for art, should be bestowed upon the cheerless walls of some hospital wards, to be a long lasting picture-gallery for a long succession of patients stretching far into the vista of the future? And above the dado hung, at intervals, oleographs and coloured prints, not garish, but truly and softly-coloured, such as will please, not weary, a child's eyes, which are forced to glance upon them through long days of lying in bed.

Tea-time is just over. The little ones that can are sitting up in their red flannel gowns, with play-tray in front of them, still finding fresh beauties in the toys provided for them. Some of the nurses are away. Two or three are busy about the ward. Sister Mercy is there, in her dark blue gown and white cap. She is tall and mother-like, kind of heart as of face, and a great favourite with the little ones. A young probationer, in her light print gown and white bands, which denote her rank, is amusing a little patient, who cannot sit up like the rest on account of some spinal affection. But suddenly little fingers and bright eyes cease to be busy with the toys, for some one is carrying a little girl into the ward. Sister Mercy goes at once to see the new patient laid down upon a bed. And Doctor Fierce, the house-surgeon, is there. Those that are old enough to understand begin to wonder what has befallen their new companion, who is to have that bed in the corner just opposite, "Little Red Riding Hood." But the screen is put around, and they know Doctor Fierce is busy. They all like Doctor Fierce. He is such a kind-hearted, good-humoured man, always saying funny things, and making them laugh even when he hurts them. He is not a bit like his name, for his eyes are blue, and there is always a smile lurking somewhere about them.

Little Elsie Inman was the new case, a little fair-haired thing, slightly made, with large blue eyes and cheeks naturally rosy, only now somewhat pale. And when the little girl had been undressed quickly and tenderly by Sister Mercy herself, Doctor Fierce examined her, and soon found out that one little leg was broken below the knee. She could not have been run over, or it must have been much more serious. As it was, the description of the case over the bed was merely inscribed, "Simple fracture of the tibia." Elsie began to come to as the doctor was finishing the bandage, and to cry for "Muvver" to deliver her from the hands of the person she imagined was hurting her so much; and she was only a bit comforted by the looks and tones of the kindly Sister. Presently Doctor Fierce went, and Elsie sobbed low, while the Sister talked to her and soothed her. In a few minutes she heard another voice near that caused her to open her eyes, tear-filled as they were, and try to wipe them with the counterpane, and look up. Yes! there was "mother," and for the time Elsie felt comforted.

Mrs. Inman had never seen the inside of a hospital before, and, like many other people, entertained the idea that it was a very dreadful place, where people who had some incurable disease were taken to have an operation performed, or when they were run over in the streets, and that they rarely came away again alive. She had half-determined in her own mind not to let Elsie stay there a minute if she could help it. But when she saw the ward, cheerful, warm, and bright, the children's faces rosy with returning health, and actually smiling, and the Sister, she was quite shaken in her intention. She did not quite know what to make of it until the Sister came forward, and, turning to Mrs. Inman, in answer to George's inquiry, said: "You're the mother of the little girl just brought in?" in a most sympathetic tone, and taking her by the hand, led her up to the bedside. Then she went on, divining the mother's first impulse.

"Oh! yes, Mrs. ('Inman,' George interposed) Inman, you may kiss her." And Mrs. Inman bent down over the bed to the little weeping face that looked up at her, and kissed it, her own eyes glistening with tears. Then she said wistfully to Sister Mercy:

"What is it, ma'am? Please tell me. I'm so anxious. Are there any bones broken? Is she much hurt?" And she began to cry in earnest.

"Don't be alarmed, Mrs. Inman," was Sister Mercy's reply; "one leg is broken below the knee, but not badly. It is strapped up now. Would you like to look at it?"

"Oh! I couldn't, I couldn't," sobbed poor Mrs. Inman.

"Come, cheer up, Tilda," said George. "You'd better see it; you'll be more satisfied."

So Mrs. Inman mustered up courage to look, as the bedclothes were turned back, at the little leg, bound round and round in its splints and bandages. She felt more consoled to think it was no worse.

"I don't think you can do better than let her stay here," said George. "What do you think, Tilda?"

Elsie's ears were pricked up at this, and she sobbed out, "Muvver 'tay here wif Elsie!"

"If you were thinking of taking her home," said the Sister, "I'm sure she'd be better here. She will get the best of attention, and after the first day or so be quite happy. She will get well sooner, and her leg will run no risk of another accident before it's quite strong."

"Well! if you think so, ma'am," said Mrs. Inman, hesitatingly, "and I can come and see her?"

"Certainly," said the Sister, "at the fixed hours; and there are a few articles we require with each patient, which you could bring with you to-morrow, for instance."

And the end of it was, that Mrs. Inman kissed Elsie again, and told her to be a good girl, and George bent down, somewhat shyly, and kissed her too, passing his fingers through her golden curls, as Elsie said:

"'Es, Elsie be dood girl. Muvver bing dolly to-morrow."

Then George and Tilda thanked the Sister and returned home, somewhat sad, yet glad to think things were no worse, and feeling drawn together closer than ever by this, the first great trouble of their married life. They both agreed that Sister Mercy was the kindest lady they had ever known. George even acknowledged to himself that his somewhat hard and sweeping views on the upper classes and carriage people might need revising, now that he had met Sister Mercy, who was distinctly one of them, and had learned that another had done those exquisite paintings in the Children's ward.

(*To be concluded.*)

